# Product Module Network Modeling and Evolution Analysis

**DOI:** 10.1155/2019/2186916

**Published:** 2019-03-06

**Authors:** Hu Qiao, Zhaohui Xu, Jiang He, Ying Xiang

**Affiliations:** ^1^School of Mechatronic Engineering, Xi'an Technological University, Xi'an 710021, Shaanxi, China; ^2^College of Mechanical and Electrical Engineering, Shaanxi University of Science and Technology, Xi'an 710021, Shaanxi, China

## Abstract

Modular technology for product design and manufacturing is an effective way to solve mass customization problems. One difficulty in the application of modular technology is that the characteristics of mass customization, such as multi batch and small batch, easily increase the complexity of the module structure of the enterprise products. To address this problem, based on complex network theory, the enterprise products module is mapped as the vertex of the network, the number of modules used is mapped as the node weight, the dependency between the modules is mapped to the edge, and the product module network is established. The brittleness risk entropy of the product module network is put forward by considering the internal and external factors that influence the application of the enterprise module to determine the rationality of the required modules' organizational structures. Then, the stability uncertainty of the product module network can be determined by calculating the brittleness risk entropy, in which the subsystem that is the most brittle risk entropy can be identified. And the evolution of the product module network can be promoted by changing factors of the entropy maximum subsystem. To analyze the change in the product module network caused by module evolution, a BBV (Barrat–Barthelemy–Vespignani) model of the product module network is established to dynamically determine the brittle risk of the product module network. Finally, the modularity structure of a series of special vehicles is used as an example to verify the presented method, and the results confirm the rationality and effectiveness of the method.

## 1. Introduction

Modular design allows enterprises to offer diversified products to respond quickly to changing market demands by improving product diversity and reduce the complexity of the engineering system. Modular technology, as the most effective and widely used technology, has become the most important feature of product structure design [1].

Under mass customization, product modules are organized and managed in accordance with certain rules, and different types of modules are collected to form the module organization structure. The modular structure of complex products has many characteristics, such as various types of modules, decentralized module sources and unbalanced use of modules. It is a complicated system that affects and interacts with many kinds of factors. A variety of factors lead to instability of the module organization structure, such as random factors, including the outsourcing module arrival failure rate and low self-made module completion rate, and objective factors, including large module demand and high module usage rates. These factors will lead to a lack of timely module supply, delay product delivery, etc., and ultimately affect the stability of production. Therefore, considering various factors, the stability of the module organization structure is improved by changing the module type, improving the module structure, developing new modules, and so on. Consequently, the study of module evolution has important significance for engineering applications.

As the complexity and type of mechanical products increase, the type and quantity of product modules increase quickly. This is an important issue in guiding module evolution. The module organization structure within an enterprise is not a simple statistic of the number of different modules. To optimize the module organization structure, it is necessary to consider a series of problems, such as the basic relationship between modules, the number of modules, and the changes in modules. The two important considerations in realizing the evolution and optimization of module organization structure are as follows:

1. How to establish the module organization structure model
2. Firstly, the module structure model must be established when researching module evolution. At present, design structure matrix [2, 3], graph theory [4], and complex network [5–7] are the main module organization structure modeling methods. Research methods describe the relationship between components and modules by establishing an undirected, directed, or weighted network model. However, most modeling objects are dependent on the product and do not consider the module organization structure of a variety of products. Using the same parts in the product family as the coincidence point and establishing multiproduct network models through product tree superposition have been proposed [8] to place products and components in the same network for research, but multilevel relationships among products, modules, components, and others coexist, which is not conducive to the evolution of research modules.
3. How to ensure the stability of the module organization structure when researching the module evolution
4. Module evolution promotes changes in the modules' organization structure, and the direction of module evolution directly affects the optimization of the module organization structure. As a complex system, the stability of module organization is critical. In the field of system stability research, Fouad [9] first proposed the concept of power system vulnerability and established a weak analysis method based on a transient energy function and artificial neural network. In 2000, Albert researched brittle sources based on a complicated theory that brought system brittleness into a new age [10]. Wang et al. [11, 12] proposed a coupled map lattice model of cascading failure that provides a good mathematical means for cascading failure models of complex networks. Jin et al. [13] proposed system brittleness with entropy theory and system mutation theory. In addition, there have recently been new advances in the theory of complex network brittleness [14, 15] and applications [16] of complex network brittleness. However, compared with other complex systems, the propagation mechanism of brittle behavior, the brittle sources, and other factors differ due to the unique characteristics of module organization structure, and the relevant mechanism needs to be analyzed in depth. In addition, the dynamics of module evolution determine the characteristics of the dynamic changes in the system. System stability, as an important indicator of system optimization, needs to follow the dynamics of updating.

In this paper, a module evolution method is proposed to improve the stability of production to solve the problem of optimizing module organization structure for modular products. Complex network theory is used to map the module organization structure as a product module network with the characteristics of a scale-free network and small-world network by analyzing the network topology characteristics of the product module network model. By analyzing factors such as the arrival rate, completion rate, and utilization rate of the product module, the brittle risk entropy of the product module network model, that is, the uncertainty of the stability of the product module network, is established. The dynamic evolution model of the product module network is established to guide changes in the product module network caused by module evolution, and thus the goal of determining the brittleness risk of the product module network dynamically can be achieved. In addition, a series of special vehicles are presented to explain the effectiveness and reasonableness of the proposed method.

## 2. Product Module Network Model

### 2.1. Product Module Network Modeling

The modular product takes the module as the core and combines different modules to form a product. The combination relationship between two modules can be mapped as an edge, and thus any modular product can be represented using a network. As a part of the product, the module needs to be assembled to form a qualified product. The module combination has a direction order based on the order of assembly. One of the main features of modular products is reusability; that is, the same module can be used for a variety of products. It is possible to research the relationship between modules of multiple products in the same network because many reusable modules are used in a series of products.

Based on the module's usage order and reuse frequency in combination, the topology of the module organization structure is formed and defined as the product module network *G* (*V*, *E*, *W*):

 
*V*: nodes set, consisting of modules
 
*E*: directed edges set
 
*W*: weight, indicating the reuse number of the module

**Figure 1:**
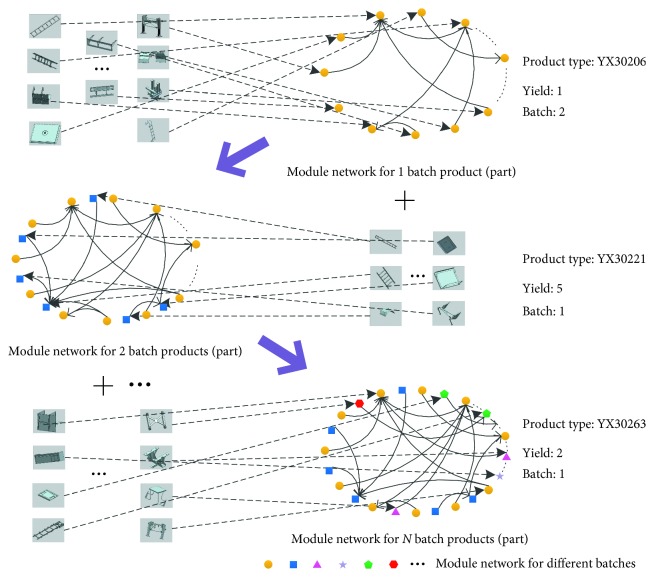
The process of product module network modeling.

A series of special vehicles designed and produced by a special vehicle manufacturer are used as an example. The product module network *G* (*V*, *E*, *W*), which contain 132 nodes and 816 edges, is constituted by 6 types and 27 product modules. As shown in Figure 1, the network comprises the modules of multiple products in accordance with the combination relation, and the direction of connection edges between nodes is determined by the assembly order of the modules.

### 2.2. Topology Analysis of the Product Module Network

**Figure 2:**
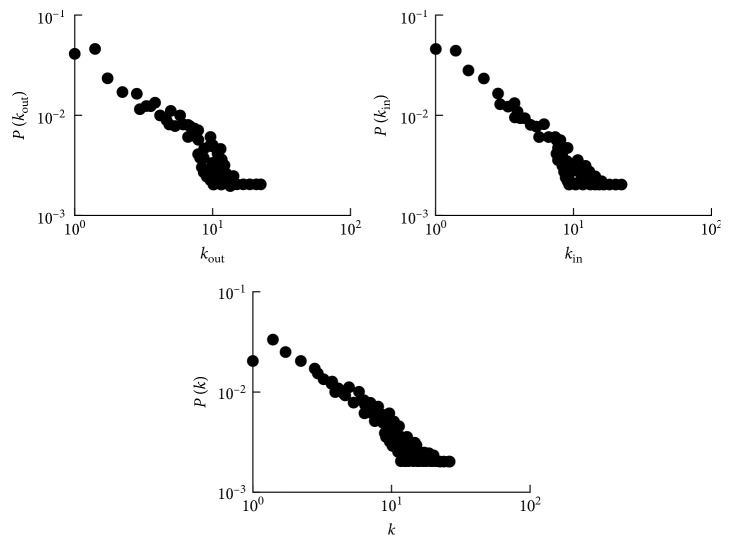
The node parameter statistical distribution of the product module network.

As one of the most basic static geometric vectors in a complex network, the degree distribution of nodes can reflect the macroscopic statistical characteristics of the network. Among the statistics of the product module network established in Section 2.1, the out-degree distribution function *P*(*k*_out_) with the change in the out-degree node *k*_out_ is drawn in a double logarithmic coordinate system, the in-degree distribution function *P*(*k*_in_) with the change in the in-degree node *k*_in_ is drawn, and the degree distribution function *P*(*k*) with the change in the degree *k* is drawn (shown in Figure 2).

Figure 2 shows that the tail of the out-degree distribution function *P* (*k*_out_) has a significant heavy-tailed phenomenon and is approximately a straight line. The whole function is consistent with the power-law distribution. The degree distribution *P* (*k*_in_) is closer to a straight line and has a significant heavy-tailed phenomenon. The degree distribution *P* (*k*) is closer to a straight line except for the initial point shifting, and the tail shows heavy tail traces consistent with the power-law distribution. Thus, the product module network conforms to the characteristics of a scale-free network, which means that the network distribution obeys an exponential distribution [17]:

(1) $$\begin{array}{c} P\left(k\right)∼k^{-r}. \end{array}$$

The average shortest distance of a random network with the same number of nodes and node averages as the product module network, which is expressed as *L*_*r*_, is calculated by formula (2) [17], which also shows the result:

(2) $$\begin{array}{c} L_{r}=\frac{\ln\;\;n}{\ln\;\;K}=2.6913. \end{array}$$

The average cluster coefficient of a random network with the same number of nodes and node averages as the product module network, which is expressed as *C*_*r*_, is calculated by formula (3) [17], which also shows the result:

(3) $$\begin{array}{c} C_{r}=\frac{K}{n}=0.0465, \end{array}$$

where *K* is the average degree of a network node.

Compared with the product module network, the average shortest path *l* (calculated by formula (2)) of the product module network is

(4) $$\begin{array}{c} l=2.9316\approx L_{r}. \end{array}$$

The average shortest path *c* (calculated by formula (3)) of the product module network is

(5) $$\begin{array}{c} c=0.3157\geq C_{r}. \end{array}$$

From formulas (4) and (5), there are a short average distance and a large average clustering coefficient in the product module network, which means that the product module network has the characteristics of a small-world network [17].

In a product module network, the out-degree reflects the module application range, the in-degree is the acceptance range of the module, the average shortest distance reflects the separation degree of the module, and the clustering factor reflects the criticality of the module. Thus, the product module network not only has the characteristics of a scale-free network and small-world network but also has some of its own characteristics:

1. In Figure 2, there are some nodes whose degree of out-degree or in-degree is 1, which indicates that these nodes can only be assembled to a specific module or only interface with a module. These modules are special modules of a single custom product, in accordance with the actual production.
2. The degree distribution of the product module network is not uniform, and there are some hub nodes. A hub node indicates a few modules that are reused by multiple products in a product module network. The reliance on these modules is excessive, which indicates that the module is less diversified or has higher market popularity. Excessive reliance can easily lead to an inability of the product module network to bear the impact of node collapse.
3. A scale-free network exhibits a high robustness to random faults and a high vulnerability to deliberate attacks. A similar situation occurs in the product module network, and therefore it is necessary to analyze the vulnerability of the product module network.

## 3. Product Module Network Brittle Analysis

For a complex system *S*, there is a subsystem or a part *S*_*i*_ that has a strong sensitivity to the environment. When *S*_*i*_ collapses because *S*_*i*_ suffers from a disturbance or attack by internal or external factors, other subsystems or parts might also collapse, potentially leading to collapse of the entire complex system. This behavioral characteristic of complex systems is called brittleness [18]. Brittleness is the basic property of complex systems that always exists and does not disappear with system evolution or environmental change.

If a module is a subsystem in a product module network, then a subsystem also has brittleness due to a low arrival rate, insufficient completion rate, and so on within the specified time. When the number of modules corresponding to the subsystem fails to support the production tasks corresponding to the product module network, the subsystem is abnormal, which is defined as subsystem collapse. The lower the effective supply rate, the greater the brittleness of the subsystem, if the module supply fault caused by subsystem brittleness is used as the characterization of the subsystem brittleness.

Assume that brittle event *I*_*x*_ in brittle event space **I** = {*I*_1_, *I*_2_, … , *I*_*X*_} that affects the arrival rate of subsystem *S*_*i*_ is a random event with probability *p*_*x*_. The module arrival rate is defined as *A*_*i*_:

(6) $$\begin{array}{c} A_{i}=1-\overset{X}{\underset{x=1}{\prod }}\left(1-p_{x}\right), \end{array}$$

where *X* denotes the total number of brittle events in brittle event space **I**, *x* *=* 1, 2, … , *X*, 0 ≤ *p*_*x*_ ≤ 1.

Assume that brittle event *J*_*y*_ in brittle event space **J** = {*J*_1_, *J*_2_, … , *J*_*Y*_} that affects the completion rate of subsystem *S*_*i*_ is a random event with probability *p*_*y*_. The module completion rate is defined as *R*_*i*_:

(7) $$\begin{array}{c} R_{i}=1-\overset{Y}{\underset{y=1}{\prod }}\left(1-p_{y}\right), \end{array}$$

where *Y* denotes the total number of brittle events in brittle event space **J**, *y* *=* 1, 2, … , *Y*, 0 ≤ *p*_*y*_ ≤ 1.

The number of modules can be ensured to meet the product module network requirements only if the outsourced module of the subsystem arrives on time and the self-made module completes on time. Therefore, the probability, (*P*_si_), that the number of modules corresponding to subsystem *S*_*i*_ satisfies the requirements of the product module network is obtained:

(8) $$\begin{array}{c} P_{\text{si}}=1-\left(1-A_{i}\right)\left(1-R_{i}\right), \end{array}$$

where *i* = 1, 2, … , *n*; *A*_*i*_ = 0, the subsystem *S*_*i*_ contains modules that are self-made; and *R*_*i*_ = 0, the subsystem *S*_*i*_ contains modules that are outsourced.

If the number of modules of system *S*_*i*_ meets the requirements of the product module network, then the internal factor, that is, the module usage rate, which leads to product module network collapse, will be considered. The higher the module usage rate *U*_*i*_, the greater the possibility of subsystem collapse due to scheduling failure, department collaboration, and other reasons.

Considering the arrival rate, completion rate, and usage rate, the brittleness measure function of subsystem *S*_*i*_ collapse caused by brittleness is defined as

(9) $$\begin{array}{c} f\left(S_{i}\right)=\frac{U_{i}}{P_{\text{si}}}, \end{array}$$

where *i* *=* 1, 2, … , *n.*

The probability of system *S* collapse is *p*_*i*_, 0 ≤ *p*_*i*_ ≤ 1, under the influence of subsystem *S*_*i*_ collapse. *p*_*i*_ is defined as the influence coefficient that the subsystem *S*_*i*_ collapse causes system *S* = {*S*_1_, *S*_2_,…, *S*_*n*_} to collapse. The normalized value of the influence coefficient and collapse probability distribution of the subsystem are calculated. The utility coefficient *q*_*i*_ of subsystem *S*_*i*_ collapse is formulated as follows:

(10) $$\begin{array}{c} q_{i}=\frac{f\left(S_{i}\right)p_{i}}{\sum_{i=1}^{n}f\left(S_{i}\right)p_{i}}, \end{array}$$

where *i* *=* 1, 2, … , *n*, 0 ≤ *q*_*i*_ ≤ 1, ∑_*i*=1_^*n*^*q*_*i*_=1.

According to Shannon theory [19], the brittle risk entropy of subsystem *S*_*i*_ can be defined as

(11) $$\begin{array}{c} G\left(S_{i}\right)=-q_{i}\text{ log }f\left(S_{i}\right), \end{array}$$

where *i* *=* 1, 2, … , *n.*

The mean value of the measure function of brittle events in the utility coefficient space is defined as the brittle risk entropy of the system, which is expressed as *H*(*S*):

(12) $$\begin{array}{c} H\left(S\right)=-\overset{n}{\underset{i=1}{\sum }}q_{i}\text{ log }f\left(S_{i}\right)=-\overset{n}{\underset{i=1}{\sum }}\left(\frac{f\left(S_{i}\right)p_{i}}{\sum f\left(S_{i}\right)p_{i}}\right)\text{log }f\left(S_{i}\right), \end{array}$$

where *i* *=* 1, 2, … , *n.*

According to the structure of the product module network, the usage rate of the module, which is expressed as *U*_*i*_, can be mapped to the ratio of the output intensity to the total output intensity of a node in the product module network:

(13) $$\begin{array}{c} U_{i}=\frac{s_{i}^{\text{out}}}{\sum s_{i}^{\text{out}}}, \end{array}$$

where *s*_*i*_^out^ = output intensity of the *i*th node, *i* *=* 1, 2, … , *n.*

The influence coefficient *p*_*i*_ of subsystem *S*_*i*_ collapse to system collapse can be mapped to the normalized value of the subsystem cluster coefficient:

(14) $$\begin{array}{c} p_{i}=\frac{c_{i}}{\sum c_{i}}, \end{array}$$

where *c*_*i*_ = the cluster coefficient of the *i*th node, *i* *=* 1, 2, … , *n.*

Thus, the brittle risk entropy of the product module network can be expressed by the following:

(15) $$\begin{array}{c} H\left(S\right)=-\overset{n}{\underset{i=1}{\sum }}q_{i}\;\log\;\;f\left(S_{i}\right)\\ =-\overset{n}{\underset{i=1}{\sum }}\left(\frac{\left(s_{i}^{\text{out}}/\sum s_{i}^{\mathrm{out}}\right)\left(c_{i}/\sum c_{i}\right)\left(1/P_{\text{si}}\right)}{\sum \left({s_{i}^{\text{out}}/\sum s_{i}^{\text{out}}}_{i}\right)\left(c_{i}/\sum c_{i}\right)\left(1/P_{\text{si}}\right)}\right)\cdot \;\;\log\frac{s_{i}^{\text{out}}}{\sum s_{i}^{\text{out}}}\frac{1}{P_{\text{si}}}, \end{array}$$

where *i* *=* 1, 2, … , *n.*

The brittle risk entropy reflects the brittle risk of the system at a moment and is the uncertainty measure of the possibility of system collapse [20]. From formula (11), the brittle risk entropy of the system is closely related to the output intensity of the network node and the node cluster coefficient. Therefore, the subsystem can be adjusted to improve the system performance and reduce brittleness risks.

Because of the different module types and sources of subsystems, there are also differences in brittle events. The uncertainty of the subsystem's brittle risk can be measured. If the risk entropy of subsystem *S*_*k*_ in system *S* is the largest, then,

(16) $$\begin{array}{c} G\left(S_{k}\right)=\max G\left(S_{i}\right), \end{array}$$

where *i* *=* 1, 2, … , *n.*

Multiple calls of formula (16) obtain several subsystems with large entropy in the system. The higher the brittle risk entropy, the poorer the grasp of the uncertainty of subsystem brittle risk. The brittle events in the subsystem with larger brittle risk entropy are analyzed to determine the main brittle factors that may lead to subsystem collapse. The subsystem brittle risk can be effectively reduced, and the system brittle risk will be reduced by controlling the main brittle factors.

## 4. Product Module Network Evolution

From formula (15), the brittleness of a subsystem comes from three aspects: arrival rate, completion rate, and usage rate. The arrival rate, completion rate, and usage rate of modules may be improved by adding a new module and deleting the old module. The brittle risk of a product module network subsystem is reduced by enhancing the control of the arrival rate, completion rate, and usage rate, gradually improving the arrival rate and completion rate and reducing the module usage rate.

In the product module network, the original network edge weight will change due to the addition of a new module. Module evolution is the evolution of the interaction between network nodes, which is consistent with the common characteristics of the actual network.

From formulas (13) and (14), the output intensity and cluster coefficients of the product module network nodes have key functions for assessing the brittle risk entropy of the system.

In the product module network, the output intensity *s*_*i*_^out^ of node *N*_*i*_ can be defined as

(17) $$\begin{array}{c} s_{i}^{\text{out}}=\underset{j\in h}{\sum }w_{i,j}, \end{array}$$

where *w*_*i*,*j*_ = the weight between two adjacent points and *h* = the number set of adjacent nodes that *N*_*i*_ points to.

Considering node *N*_*i*_ and the weight between two adjacent points that *N*_*i*_ points to, the output intensity *s*_*i*_^out^ can reflect the influence of *N*_*i*_. The output intensity of nodes indicates the usage frequency of *N*_*i*_ in the product module network.

The cluster coefficient *c*_*i*_ of the *i*th node in the product module network is

(18) $$\begin{array}{c} c_{i}=\frac{1}{s_{i}\left(k_{i}-1\right)}\cdot \underset{j,k}{\sum }\frac{\left(w_{i,j}+w_{j,k}\right)}{2}\left(a_{ij}a_{jk}a_{ki}\right), \end{array}$$

where *s*_*i*_ = the point intensity of *N*_*i*_ and *k*_*i*_ = the degree of *N*_*i*_.

The cluster coefficient *c*_*i*_ is the weight relationship between the node and its surrounding nodes and can comprehensively reflect the importance of the node. In a product module network, a node's cluster coefficient expresses the importance of modules contained in the nodes.

According to formulas (17) and (18), the output intensity and cluster coefficient of the network node are calculated based on the edge weight. To adapt to the dynamic evolution process of the module, it is necessary to analyze the product module network dynamically.

The BBV (Barrat–Barthelemy–Vespignani) model is a network evolution model based on the point intensity driving and weight strengthening mechanisms. It can imitate changes in interaction intensity in a real system [21]. Here, the BBV model is used to describe and analyze the evolution of the product module network in a dynamic environment.

In a product module network that contains *n* nodes, when new node *N*_*j*_ is added to the network in a point-driven manner at each time interval, the weights of old node *N*_*i*_ and new node *N*_*j*_ will be allocated as

(19) $$\begin{array}{c} P_{j⟶i}=\frac{s_{i}}{\sum s_{j}},\\ w_{i,j}\left|{}_{\text{new}}\right.=w_{i,j}+\delta \frac{w_{i,j}}{s_{i}}, \end{array}$$

where *s*_*i*_ = the point intensity of *N*_*i*_ and *δ* = weight increment.

When *N*_*j*_ increases, the weight of *N*_*j*_ will have an increment *δ*. *δ* will be divided into all nodes connected to *N*_*i*_ based on weight.

The output intensity of *N*_*i*_ in the product module network can be modified as

(20) $$\begin{array}{c} s_{i}^{\text{out}}=\underset{j\in h}{\sum }w_{i,j}\left|{}_{\text{new}}\right.. \end{array}$$

The cluster coefficient of the *i*th node can be modified as

(21) $$\begin{array}{c} C_{i}=C_{i}\left|{}_{\text{new}}\right., \end{array}$$

where *C*_*i*_|_new_ = the new cluster coefficient after weight change.

The objective function of solving the subsystem with the maximum entropy can be modified to the following:

(22) $$\begin{array}{c} G\left(S_{k}\right)=\max G\left(S_{i}\right)\left|{}_{\text{new}}\right., \end{array}$$

where *i* *=* *j*, 1, 2, … , *n* and *G*(*S*_*i*_)|_new_ = new subsystem risk entropy after weight change.

Then, the change in the brittle risk entropy of the system is shown as

(23) $$\begin{array}{c} H\left(S\right)=-\overset{n}{\underset{i=1}{\sum }}q_{i}\text{ log }f\left(S_{i}\right)\left|{}_{\text{new}}\right., \end{array}$$

where *i* *=* *j*, 1, 2, … , *n.*

The new node weight information after network evolution can be obtained by using the BBV model, and the BBV model can adapt to the dynamic environment of the network evolution, dynamically determine which subsystem has the maximum brittle risk entropy in the product module network, and improve the grasp of the brittle risk of the product module network. In addition, the BBV model provides support for determining the brittle events of the subsystem and reducing brittle risk during network evolution.

## 5. Case

A series of special vehicles designed and produced by a special vehicle's manufacturer were tracked for 6 months using the above methods. The first 6 months of production tasks without this method were counted and analyzed according to production records. The effectiveness of the method was verified by comparing the effects before and after the application.

**Figure 3:**
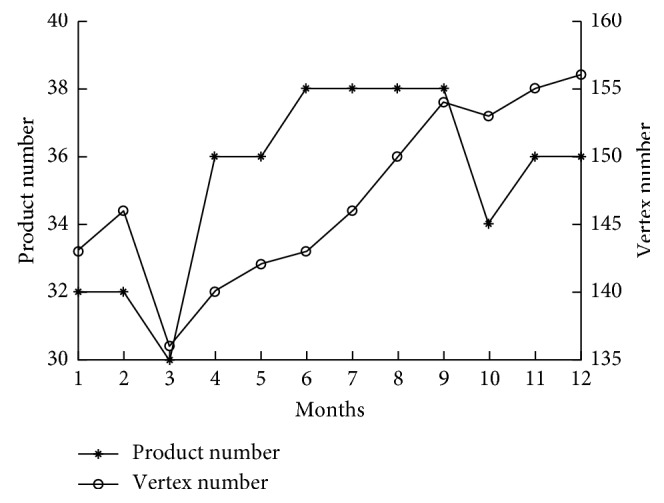
The output and module type in every month.

The monthly output in a production year and the node number changes of the product module network were obtained as shown in Figure 3.

In a production year, the product output of this series maintains 30–38 units/month. The maximum number of used modules is 156, and the minimum number is 136. Module types vary with monthly production in every month.

**Figure 4:**
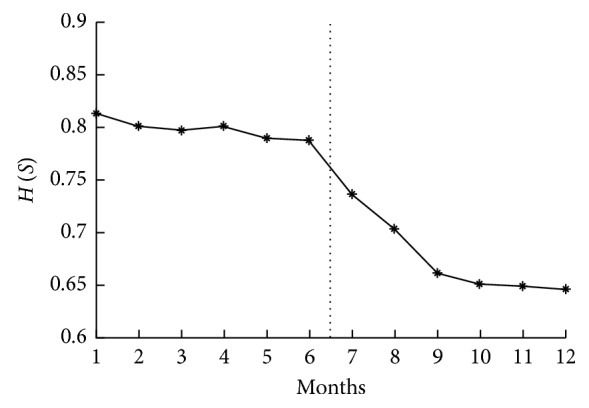
The brittle risk entropy of the product module network.

The brittle risk entropy of the first 6 months of the product module network is calculated by formula (15). Starting in the 7th month, the impact of the arrival rate, completion rate, and usage rate on subsystems with large entropy values is analyzed to promote and realize the evolution of the product module network by the method shown in Section 4, and the brittle risk entropy of the product module network in the next month is calculated. Finally, the brittle risk entropy of the product module network in a production year can be obtained, as shown in Figure 4.

The trend of the brittle risk entropy of the product module network shows that the brittle risk of the product module network declines before using this method (from the 1st to 6th month), with a maximum of 0.8136 and a minimum of 0.7880. However, the rate of decline is relatively slow, and there is even a slight increase in the 4th month.

Starting from the seventh month, the brittle risk entropy function of the subsystem is used to locate a subsystem with larger brittle risk entropy, and module evolution is performed based on the brittle event type. The brittle risk entropy of the product module network in the 7th, 8th, and 9th months declines rapidly from 0.7880 to 0.6616 because the subsystem with higher risk entropy is located precisely. This downward trend continues in the 10th, 11th, and 12th months.

Compared with Figures 3 and 4, the brittle risk entropy trend of the product module network in the first six months is consistent with the monthly output trend and the module type usage trend, which shows that the original brittle risk control is low. In the 4th month, there is high monthly output and large brittle risk entropy of the product module network. According to the production record, the number of product module changes increases from 5 in the 3rd month to 8 in the 4th month, which indicates that it is difficult for the product module network to resist the risk from production task changes.

From the 4th month to the 9th month, the monthly production tasks include 8 models. From the 4th month to the 6th month, the number of module types increases from 140 to 143, and the brittle risk entropy of the product module network decreases from 0.8012 to 0.7880. However, after introducing complex network theory, the number of module types increases from 143 in the 6th month to 154 in the 9th month, and the brittle risk entropy of the product module network decreases from 0.7880 to 0.6616. In addition, the evolution speed of the module is obviously improved, and the brittle risk entropy of the product module network is reduced rapidly, which indicates that the control ability of the product module network against brittle risk is enhanced.

The brittle risk entropy of the product module network slows in the 10th month, but there is a downward trend. According to production records, the number of product types decreased from 8 to 7 in the 10th month, but a new model was introduced. The brittle risk uncertainty of a new module may increase the brittle risk uncertainty of the product module network, which leads to slowing of the brittle risk entropy of the product module network. A similar phenomenon is observed in the 11th to 12th month, indicating a significant increase in the brittle risk resistance of the product module network and that a small number of external modules have a limited influence on the robustness of the product module network after effectively reducing the brittle risk entropy of the product module network.

Therefore, the effectiveness of the module evolution method based on brittle risk entropy analysis of the product module network is validated.

## 6. Conclusions

It is important to improve enterprise risk resistance and increase product diversity by optimizing module organization structure in production enterprises using modular technology. As a basic part of the product, there is a necessary relationship between the modules. As the product continues to be produced, the module usage of the product continues to accumulate. According to the relationship between modules and the accumulation of modules, a product module network can be established to study the module organization structure of the enterprise. Accordingly, the development law of the organizational structure of the enterprise module can be obtained to realize the optimization of the enterprise module organization structure.

In this paper, a product module network is built based on complex network theory. The network has small-world character and scale-free character according to an analysis of the topology characteristics of the network and shows uneven use of specific modules. The subsystem in the network with the maximum brittle risk entropy is obtained by establishing the brittle risk entropy function. Considering the brittle event source that leads to subsystem brittle risk, module evolution is put forward to reduce the brittle risk entropy of the product module network, which shows that the ability to grasp the brittle risk of the network is improved. The BBV model promotes product module network evolution, and the change in the brittle risk entropy of the product module network can be grasped dynamically.

The product module network is built based on a series of products that use some of the same modules. The product module network can be set up separately to analyze the module organization structure in enterprises with multiple product lines. If all products of the enterprise have some of the same modules, then a full product module network can be built and the module structure is analyzed uniformly. The product module network of the whole product series may be large, which will affect the calculation efficiency and optimization effect because the construction of the product module network is based on the types of modules and the number of reused modules.

## Data Availability

All data used in this manuscript come from a Chinese tank-truck manufacturer company. But according to the cooperation agreement, real data to the third part cannot be provided. If anyone who needs data to confirm the methods in this manuscript, please contact the corresponding author.
